# Influence of α-Particle Radiation on Intercellular Communication Networks of Tunneling Nanotubes in U87 Glioblastoma Cells

**DOI:** 10.3389/fonc.2020.01691

**Published:** 2020-09-04

**Authors:** Nicole Matejka, Judith Reindl

**Affiliations:** Institut für Angewandte Physik und Messtechnik, Fakultaet für Luft- und Raumfahrttechnik, Universitaet der Bundeswehr Muenchen, Neubiberg, Germany

**Keywords:** cellular communication, tunneling nanotubes, high-LET, cancer, bystander effect, glioblastoma

## Abstract

Cellular communication plays a crucial role in the coordination and organization of cancer cells. Especially processes such as uncontrolled cell growth, invasion, and therapy resistance (development), which are features of very malignant tumors like glioblastomas, are supported by an efficient cell-to-cell communication in the tumor environment. One powerful way for cells to communicate are tunneling nanotubes (TNTs). These tiny membrane tunnels interconnect cells over long distances and serve as highways for information exchange between distant cells. Here, we study the response of cellular communication via TNTs in U87 glioblastoma cells to homogeneous irradiation with α-particles as a stress factor. We describe the development of TNT networks in certain time steps after irradiation using confocal live-cell imaging and suggest an evaluation method to characterize these communication networks. Our results show that irradiated cells establish their network faster and have more cell-to-cell connections with high TNT content than sham-irradiated controls within the first 24 h. These findings suggest that there is an additional trigger upon radiation damage which results in fast and intensive network formation by TNTs as a radiation damage response mechanism.

## Introduction

Glioblastomas are one of the most common and most aggressive brain tumors, which are characterized by their high invasiveness and recurrence. Despite multimodal treatment, patients have a median survival of no more than 15 months and show a five-year survival rate below 10% (1–3). This poor prognosis is a result of the aggressive nature of glioblastomas composed of genomic instability, uncontrolled cellular proliferation, intratumoral heterogeneity, resistance to apoptosis, and high diffuse infiltration rates into the surrounding tissue (4–7). Due to these features, glioblastomas exhibit a considerably high chemo- and radioresistance, and despite extensive research on glioblastoma treatment, the responsible mechanisms for the aggressive nature are poorly understood or even unknown.

Radiotherapy is, besides surgery and chemotherapy, mostly in combination with one or even both, the treatment of choice for glioblastoma for ∼50% of all treated tumors worldwide (8, 9). The aim of radiotherapy is to specifically exploit the harmful effects of radiation in order to stop the proliferation of tumor cells but to protect healthy tissue as much as possible at the same time. For this purpose, it is indispensable to comprehend how radiation affects tissues and organisms and to understand the principle mechanisms occurring in cells upon radiative exposure. From a molecular biological point of view, ionizing radiation affects cellular life by depositing energy in cells, which causes breakages of chemical bonds. Therefore, proteins, lipids, genetic material, as well as other cellular components can be damaged by radiation. A critical damage for the survival of cells is the DNA double strand break (DSB), in which the DNA, the carrier of the genomic information, is completely severed (10). An erroneous repair of this type of damage can lead to cell death or mutation and consequent tumor formation. However, in cellular networks such as tissues, not only DNA damage in the single cells but also intracellular signal transduction as well as cell-to-cell communication play key roles in the damage response. It has been observed that irradiated cells send signals to neighboring cells, thus influencing the cellular survival of these cells, too. This communication can lead to so-called non-targeted effects such as the bystander effect, in which non-irradiated cells show biological radiation response due to signal-transfer from neighboring, irradiated cells (11, 12). In contrast, it was also reported that healthy cells can transport organelles, proteins, or signals to damaged cells in order to support repair and cell survival (13–16). In both cases, cell-to-cell communication directly influences the biological effects to the tissue and therefore to the organism caused by radiative stress. The underlying mechanism as well as the question of to what extend cellular communication affects the cell survival and genetic alterations after irradiation remain obscure (17). During evolution, cells developed several approaches to communicate. In 2004, a new kind of intercellular communication was reported and termed tunneling nanotubes (TNTs) (18). TNTs are thin membrane channels with a diameter in the nanometer range that directly connect cells over long distances up to 100 μm (19). They facilitate the direct cell-to-cell transfer of several cargoes such as organelles, viruses, and signals (20). Membranous connections between cells are not only found *in vitro*; such communication networks also occur *in vivo* (16, 21, 22). It was shown that especially in glioblastomas, membrane tunnels can form complex communication networks which have several biological functions and are responsible for enhancing tumor progression, radio- as well as chemoresistance (23). Furthermore, TNTs are more frequently found under a wide range of stress conditions including hypoxia (24, 25), serum starvation (22), infection (26), inflammation (21, 27), toxic treatment (28), UV- (15), and X-ray- (16), and particle-irradiation (29). Thus, it is strongly suspected that TNTs are highly linked to stress response and are triggered by stress alarm signals. For these reasons, cellular communication along these versatile, flexible membrane bridges might be a promising target for cancer treatment, especially for highly migratory and invasive tumors like glioblastomas which have a poor prognosis. A better understanding of the direct cellular response to radiation via TNTs might help to improve radiation therapies. New therapy approaches can be developed which influence the transfer of signals or the network itself. These drugs may be able to amplify the cell killing effect in the tumor environment. Also rescue of damaged healthy tissue can be a target of this kind of new therapy approaches.

Here, we study the response of TNT communication networks in glioblastoma cells on radiative stress induced by α-particle radiation. In this context, two essential questions are addressed: whether TNT communication networks are indeed influenced by particle radiation and if cellular communication is enhanced due to radiation exposure. Furthermore, we were interested in characterizing the complexity and strength of the cellular network formed by TNTs. We therefore developed an analysis method for TNT networks *in vitro* for a quantitative analysis of cellular communication via TNTs. Here, the TNT network is analyzed by addressing parameters regarding cell-to-cell connectivity and TNT density within one connection. Cells are classified into *isolated* cells, which are not involved in the network, and *connected* cells, which contribute to the network. Cell-to-cell connections are subdivided into *simple* and *complex* connections with respect to the number of TNTs they consist of in order to dissolve the strictness of the individual connections. With this method, it is possible to comprehend direct cellular communication response to radiation and to gain insight into the influence of cell-to-cell communication on the survival of cells and their behavior upon radiation.

## Materials and Methods

### Cell Culture and Irradiation

The human U87 (ATCC, HTB-14) glioblastoma cell line was kindly provided by the Institute for Radiation Medicine (Helmholtz Zentrum München GmbH, 85764 Neuherberg, Germany) and cultured in DMEM, high glucose medium (Sigma-Aldrich) supplemented with 10% FCS and 1% Penicillin/Streptavidin at a temperature of 37°C (100% humidity, 5% CO_2_). One day before irradiation, cells were seeded on round, high precision glass coverslips with 25 mm in diameter and a precise thickness of 170 ± 5 μm (Marienfeld, 150,000 cells/well). The cells were irradiated by α-particles using an Americium-241 source with an activity of 0.37 GBq, resulting in a dose rate of 0.12 Gy/min. The irradiator was built and calibrated by Roos and Kellerer (30) and ensures a homogenous dose distribution. We did further calibration using CR39 nuclear track detectors to precisely get the dose rate of 0.12 Gy/min (7). The functionality is ensured by measurements using dosimeters during the whole irradiation period. When reaching the cell layer, the α-particles have a reduced energy of 1.4 MeV which corresponds to a LET of 200 keV/μm. The cells were irradiated for 10 minutes resulting in a final dose of 1.2 Gy. This was the maximum possible dose for irradiation. Cells needed to be irradiated without medium coverage, and at 10 min the layer was reduced to zero (see Supplementary Figure 1). If cells would be kept longer, they would dry out and cell death would occur. The used dose is comparable to the dose of 1.3 Gy, which was used in a previous study, where cell survival and invasion of glioblastoma was studied using α-particle radiation (7). After irradiation, the cells were further cultured in fresh medium at 37°C, 100% humidity, and 5% CO_2_ until evaluation. The experiment was conducted 3–4 times with one sample each. At 72 h for the sham-irradiated control, only two samples worked.

### Plasma Membrane Staining and Live-Cell Confocal Imaging

After post-irradiation incubation of 1, 6, 24, and 72 h, the cells were labeled with a 1.5X CellMask Orange plasma membrane stain solution (Thermo Fisher Scientific) for 10 min at 37°C, 100% humidity, and 5% CO_2_, resulting in a stable, homogeneous fluorescence labeling of the plasma membrane in living cells.

For live-cell confocal imaging, a custom-made live-cell imaging container and a confocal microscope (Leica TCS SP8 3X) were used. Sample, microscope stage, and microscope were kept at a constant temperature of 37°C by a climate chamber. The excitation laser wavelength for CellMask Orange was 554 nm, with a detection range of 567–635 nm. Laser power for the excitation laser was in the range of 5 mW. In order to record a large area with best resolution, mosaic images were acquired using a 100× oil objective (Leica HCX PL APO 100x/1.4 Oil), resulting in a lateral resolution of 250 nm and an axial resolution of about 600 nm. Per sample, 100 partial images with an overlap of 20% were acquired and collected together to create one final merged image. This image has a size of about 670 μm × 670 μm. Each final merged image contains between 30 and 200 cells per sample. All samples were acquired in z-stacks with a step size of 400 nm and a pixel size of 40 nm. Live-cell imaging was preferred to cell fixation in order to avoid TNT breakage and distortion (18). The cells were scanned bidirectionally and with a scanning speed of 600 Hz to ensure a fast image acquisition, which reduces movement artifacts and stressing of the cells caused by long light exposures. Additionally, the complete image acquisition duration was kept under 1 h to ensure as few network changes as possible during image capture.

### Statistical Analysis

For resolving significant differences, the two-sample t-test (GraphPad QuickCalcs) was used and a *p*-value of ≤ 0.05 was considered statistically significant.

## Results

To our experience, TNTs can be formed at any time in a cell culture. However, the amount of TNTs or rather the cell-to-cell connectivity established by TNTs can vary and depends on the current stress level of the cells. One aim of this pilot study was to design a network analysis method, which enables researchers to trace the development of a TNT communication network in living cells. We applied this method on α-particle irradiated cells. Irradiation was performed using 1.2 Gy, which was the highest dose possible with this imaging setup and is comparable to the dose used in previous studies on the reaction of glioblastoma to α-particle radiation (7).

### Network Analysis Method

**FIGURE 1:**
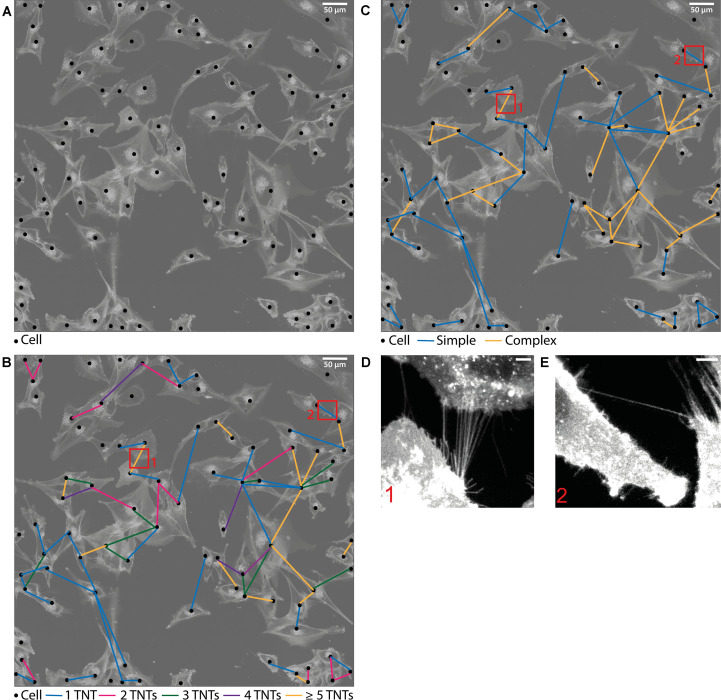
Evaluation method of the TNT network. **(A)** Drawn picture of one sample. The original image is transparent in the background where the cells are visible as white structures. Each cell is marked as a black dot. **(B,C)** TNT connections between the cells are drawn as colored lines. The colors represent the different tube density per connection. Connections containing 1–2 TNTs are referred to as simple connections whereas connections consisting of more than 2 TNTs are referred to as complex connections. The enlargements correspond to the respective selection indicated with red frames, showing a dense tube connection **(D)** and a single tube connection **(E)**. These two images are maximum projections of the corresponding confocal z-stacks. Scale bars: **(A–C)** 50 μm, **(D,E)** 5 μm.

The analysis method can be followed in Figure 1: In the first step of the evaluation, each cell was located and marked by a black dot in a transparent copy of the respective sample (Figure 1A). Here, the original picture is faded in the background and the cells are visible as bright structures. In the second step, the TNT connections between the cells were tracked and the determined number of connections was drawn as a color-coded line in the respective transparent copy (Figures 1B,C). TNTs were counted by hand while scrolling through the image and looking at each cell separately. For evaluation, it was distinguished whether the cells were connected by 1–2 or more than 2 tubes. Connections containing 1 or 2 tubes are referred to as *simple* connections, whereas those consisting of 3 or more tubes are referred to as *complex* connections (Figure 1B). The underlying idea of the differentiation of connections according to the tube density is that the exchange of signals and cargoes is enhanced when more tubes are available for the transport (31, 32). Thus, it reveals the strength of the individual connection. A partition into more than two subgroups, e.g., each TNT number alone, was not recommended because connections containing exactly 2, 3, of 4 tubes were rarely present (see Figure 1C and Supplementary Table 1) independent of treatment and time. Furthermore, at a TNT number of 5 or higher, the individual TNTs inside the connections can be so close together that they are not distinguishable anymore and, therefore, not countable. Thus, a classification of connections into two (*simple* and *complex*) instead of more subgroups was used for a quantitative evaluation. Maximum projections of a very dense tube connection and a single tube connection are shown in Figures 1D,E, respectively. These are the corresponding enlargements of the selections marked by red frames in Figures 1B,C.

With this method, the connection frequency per cell, subdivided into the corresponding tube density within the connection, can be determined. This is done by counting the respective colored lines. Additionally, one can identify how dense the cells are connected among each other. This cell-to-cell connectivity can be studied by counting the connected lines at each dot, in other words, by counting the number of cells to which the currently viewed cell is connected. With these measured variables it is possible to make qualitative and quantitative statements of the cellular communication systems composed of TNTs.

The TNT networks were evaluated at several times in order to comprehend possible communication stages during the recovery phase after irradiation. Finally, the irradiated samples were compared to sham-irradiated controls as reference to establish the impact of radiation on TNTs.

### Temporal Development of Cell-to-Cell Connectivity

**FIGURE 2:**
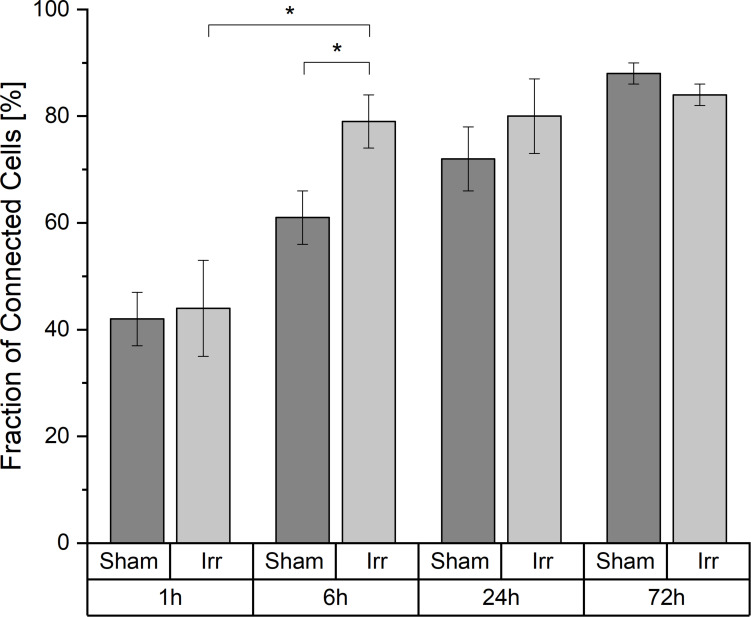
Temporal development of the cell-to-cell connectivity. Mean values ± SEM are shown. A *p*-value ≤ 0.05 is indicated by *.

A cell is considered a connected cell if it has at least one connection. With growing time, the fraction of connected cells increases significantly in both groups, irradiated and non-irradiated samples (Figure 2). At 1 h after irradiation, both groups have the same quantity of connected cells (44% ± 9% in irradiated and 42% ± 5% in sham-irradiated cell populations). After the total observation time of 72 h, the fraction of connected cells increases to values of (84 ± 2)% in irradiated cell populations and (88 ± 2)% in sham-irradiated controls. However, it is also recognizable that this development happens with different speeds in the respective cell populations. Irradiated cell populations increase their fraction of interconnected cells faster than sham-irradiated control populations. Six hours after irradiation, a significant difference (*p* < 0.05) between the fractions of interconnected cells of irradiated (79 ± 5%) and sham-irradiated controls (61 ± 5%) is visible. Irradiated cell populations show almost twice as many connected cells (79 ± 5%) compared to 1 h after irradiation (44 ± 9%). After this jump within the first 6 h after irradiation, this value does not change much during the remaining observation time, staying at a fraction of about 85% of all cells that are connected to at least one other cell. In contrast, the fraction of connected cells in sham-irradiated controls seems to grow more continuously and not volatile. After 24 h, the difference between irradiated and sham-irradiated cells is not significant anymore. After 72 h, the connectivity of both groups equaled completely to the level of about 85% connected cells.

**FIGURE 3:**
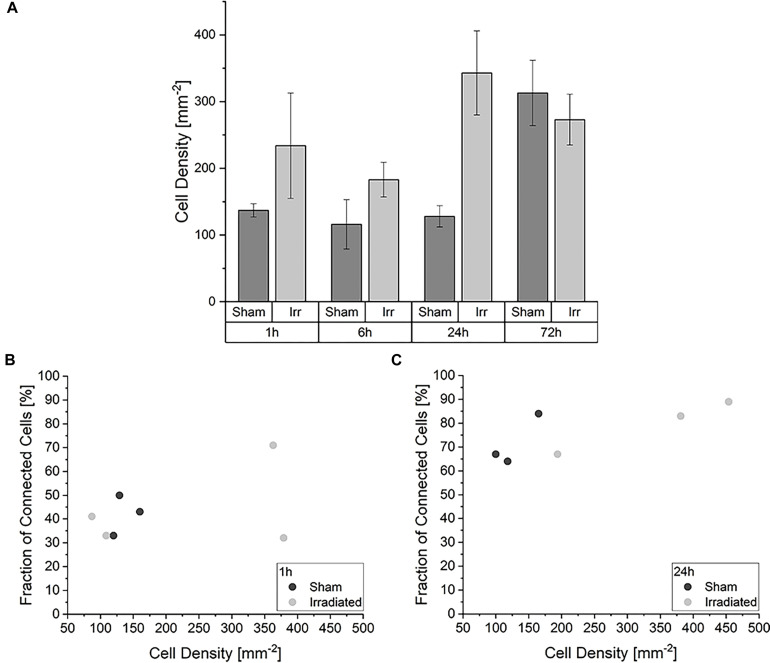
Impact of the cell density in the development of the TNT network. **(A)** Average cell densities of sham- and irradiated samples at different incubation times. Mean values ± SEM are shown. Analysis of the fraction of connected cells within irradiated and sham-irradiated samples in dependence of the cell density for the times 1 h **(B)** and 24 h **(C)**. Each dot represents one sample.

When considering this development, we asked ourselves whether different cell densities play a role in the characteristic of the TNT network. In Figure 3A the average cell densities for both groups at the four incubation times are shown. In contrast to the temporal development of the cell-to-cell connectivity (Figure 2), there is no noticeable steady growth of the cell density over time. The average cell densities for sham and irradiated samples at one time-point are comparable, except for 24 h. Here, the cell density of the irradiated samples is much higher than that of the sham-irradiated controls. For a detailed analysis, we directly looked at the single samples. For comparison, the cell density for each sample for 1 h is shown in Figure 3B and for 24 h in Figure 3C. The 6 and 72 h are shown in Supplementary Figure 2. One hour after (sham-) irradiation, the sham-irradiated controls all have a similar cell density, whereas the irradiated samples have two low and two high cell densities. However, in the two samples with a higher cell density no trend toward a higher cell-to-cell connectivity is visible, as one sample has a low fraction and one a high fraction of connected cells. For 24 h the effect that the cell density does not play a specific role in the cell-to-cell connectivity is even more pronounced. Here, all samples show similar fractions of connected cells independent of irradiation status and cell density. Overall, the cell densities of the individual samples differ from one another, but there is no concrete correlation between cell density and fraction of connected cells identifiable. Samples with a higher cell density do not show a significant rise of the cell-to-cell connectivity. Only with growing time the fraction of connected cells steadily increases, traceable in Figure 2 and when comparing the fraction of connected cells at 1 h (Figure 3B) and 24 h (Figure 3C), where the cell-to-cell connectivity grows from 43 to 81%, respectively.

### Temporal Development of Complexity of the Connections

**FIGURE 4:**
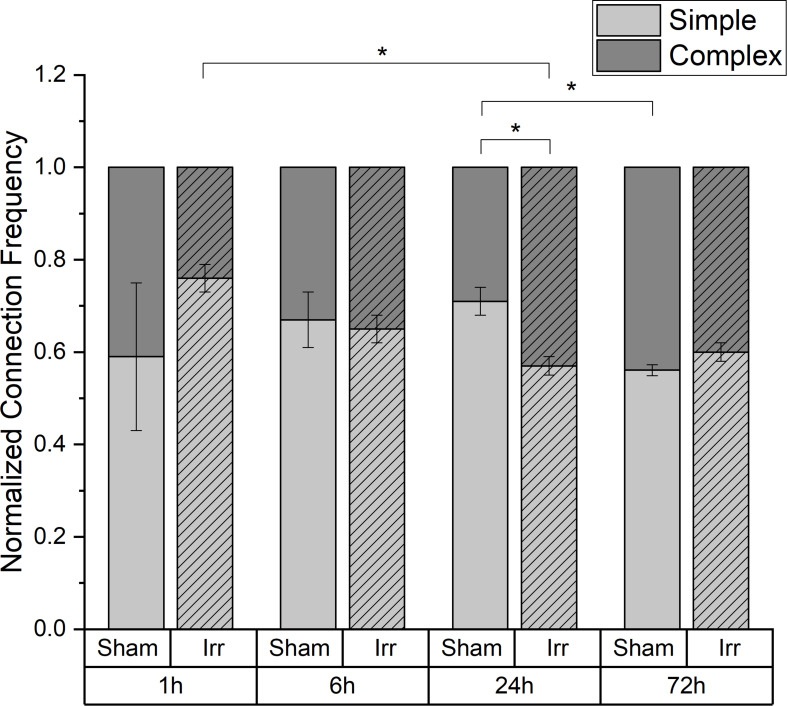
Temporal development of the proportion of simple and complex connections in irradiated and sham-irradiated cell populations. Those frequencies are normalized to the total number of found connections. Mean values ± SEM are shown. A *p*-value ≤ 0.05 is indicated by *.

In Figure 4, the temporal development of the distribution between the two kinds of connection, *simple* and *complex*, for irradiated and sham-irradiated samples is shown. The frequencies are normalized to the overall number of connections found in the respective sample. Regardless of time and treatment, there are always more *simple* than *complex* connections. However, the exact distribution of the different connection types is neither independent of time nor treatment. Up to 6 h after (sham-) irradiation, the partitioning of the connections is the same for irradiated and sham-treated samples. Thereby, an average frequency of 0.66 ± 0.01 for *simple* connections and 0.34 ± 0.01 for *complex* connections was observed. However, at an incubation period of 24 h, there are significant differences regarding the proportion of simple and complex connections: Non-irradiated cells exhibit much more *simple* connections than irradiated cells (*p* < 0.05). Consequently, more *complex* connections are found in the irradiated samples than in the control samples. After three days, the proportions for irradiated and sham samples converge again. In the irradiated cell populations, the proportion of *complex* connections with more than two TNTs and therefore network strength increases significantly from 1 to 24 h after irradiation, remaining at this level for the next two days until the end of incubation. In sham-irradiated samples, the complexity of connections is largely constant over the first 24 h and then increases significantly in the following 48 h.

### Temporal Development of the Fraction of Highly Connected Cells

**FIGURE 5:**
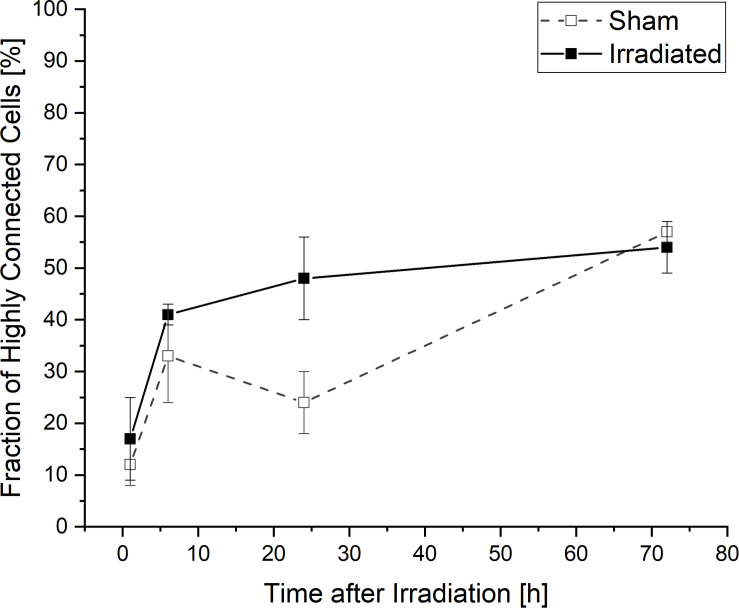
Temporal development of highly connected cells which are interconnected to two or more cells. Mean values ± SEM are shown.

When considering the temporal development of the fraction of cells which are at least connected to two or more cells and thus highly connected into the network, a lower connectivity is recognizable for sham-irradiated controls compared to irradiated samples only at 24 h after (sham-) irradiation (Figure 5). In 1 and 6 h after (sham-) irradiation, irradiated and sham-irradiated samples exhibit nearly the same proportion of highly connected cells. This behavior changes at an incubation time of 24 h. At this time, the amount of highly connected cells increases further in irradiated cell populations to a value of 48 ± 16%, whereas untreated cell populations seem to exhibit a small decrease of this cell fraction to 24 ± 12%. This difference vanishes again after 72 h, where the fractions of highly connected cells align with each other, reaching values of 54 ± 5% and 57 ± 2% in irradiated and sham-irradiated cells, respectively. During the complete observation time, the number of highly connected cells increases in both groups, sham- and irradiated.

## Discussion

The presented data show that both irradiated and non-irradiated cells expand and upgrade their communication network during growth, visible by more *complex* connections, i.e., higher number of TNTs per connection, and higher cell-to-cell connectivity, i.e., more cells connected to at least one other cell as well as more cells connected to several other cells, 72 h after (sham-)irradiation. However, irradiated cells establish their TNT communication network faster than sham-irradiated cells. The fraction of cells that are connected to at least one other cell and thus involved in the network jumps to a higher level in irradiated samples than in sham controls between 1 and 6 h after irradiation. Sham-irradiated cell populations show a more continuous network growth and the same connectivity of about 85% that irradiated samples show at 6 h are only reached after about 24 h.

These findings suggest that there are different triggers inducing the TNT formation in irradiated and non-irradiated cells. It seems that in irradiated cells, the TNT formation and the development of the cellular communication network is accelerated by an additional mechanism, which is not active in the sham-irradiated cell populations. With this faster development of their TNT communication network, irradiated cells may be able to deal with the radiative stress and to trigger survival mechanisms.

TNT formation can be realized by cell dislodgement after cell-to-cell contact or by filopodia growth (33). We have observed and recorded that TNT formation in U87 cells occurs via cell dislodgement (Supplementary Figure 3), but this does not exclude that TNT formation can additionally be realized by filopodia growth in U87 cells. It might be possible that selective TNT formation is realized by filopodia growth and the usual communication network is established by cell dislodgement after cell division or encountering of cells. Therefore, it could be possible that the communication network in cells is enhanced upon irradiation and an additional mechanism causes an increased triggering of TNT formation by filopodia growth. This would explain the immediate rise of the fraction of interconnected cells within 6 h in irradiated cell populations. The release of stress signals into the medium, originating from the irradiated, stressed cells, could induce filopodia growth and lead to an orientated TNT formation.

After this immediate jump, the networking cell fraction of about 85% does not change further in irradiated cell populations during the complete observation period of up to 72 h, suggesting that after 6 h the additional triggering of TNT formation is attenuated or a saturation regarding the development of the TNT network has been reached.

In almost all cellular communication networks there are cells that are interconnected with several cells, i.e., more than one other cell. This portion of cells can be considered as an indicator of the complexity of a communication network. Here, more highly connected cells tend to be present in irradiated samples than in controls after 24 h. Additionally, there are significantly more *complex* connections found in irradiated cells than in sham-irradiated controls at this point of time. After the expansion of the TNT network within the first 6 h by involving as many cells as possible, the focus is now on condensing and strengthening their network. By contrast, the sham-irradiated samples build their network more slowly and are non-complex, mostly by the formation of *simple* tube connections to one other cell.

After three days, irradiated and sham-irradiated samples have aligned themselves and exhibit the same values regarding fraction of connected cells, average and distribution of the number of connections per cell (Supplementary Figure 4 and Supplementary Table 1), as well as proportion of *simple* and *complex* connections. This suggests that the additional triggering of TNT formation in irradiated cells has been stopped and the network is not further expanded and strengthened by the irradiated cells at a certain point. This might be due to a saturation in the establishment of the TNT network or because irradiated cells are no longer able to further expand their communication network. Furthermore, the fact that most repair processes (about 88% γ-H2AX fluorescence decay) are finished 48 h after high-LET irradiation (34) can lead to a stop of oriented TNT formation. Additionally, remaining TNT connections to apoptotic cells, which cannot be rescued, might become detached to isolate these irrecoverable cells, similar to the model proposed by Rustom (35). Consequently, it would be interesting to evaluate incubation times longer than three days, to figure out if the controls are further able to expand and strengthen their network and thus will pass the irradiated cells. If this is true, it would demonstrate that cells are hampered in their communication via TNTs by irradiation.

Overall, the aim of this small sample pilot study was to determine whether there are any differences in cell communication by TNTs between irradiated and non-irradiated cells. The findings demonstrate that the communication network via TNTs is influenced by irradiation and its establishment is accelerated. Irradiated cells build up and condense their communication network faster than non-irradiated cells within the first 24 h. However, after this period the controls catch up and are equal again within 72 h after (sham-) irradiation.

With our analysis method for investigating TNT networks, it is possible to follow and draw quantitative conclusions about the cellular communication along these tiny tunnels. In the literature, scientists often count each individual TNT and define parameters like the “TNT index,” which gives the number of TNT per cell (36, 37). However, when considering glioblastoma cells such as U87 cells, which can have very dense cell-to-cell connections consisting of many indistinguishable TNTs, counting of each individual TNT is sometimes even impossible. Additionally, it has been reported that a higher number of TNTs between two cells leads to an amplification of electrical signals (31, 32). Thus, one can assume that a cell-to-cell connection with a higher TNT density is probably more efficient for exchanging many cargoes in a short period than a connection by only a few TNTs. In this context, we think that a differentiation between cell-to-cell connections with a high or low TNT density is more appropriate than counting each individual TNT.

A major challenge in investigating the response of cellular communication via TNTs to stress occurs due to the fluctuations of cell density. Since TNTs can be formed after direct cell-to-cell contact, one could imagine that more TNTs can be found in denser cell populations than in non-confluent. In this study, U87 cells were cultivated for 72 h, leading to a higher cell density due to growth compared to the cell density directly after irradiation. Also seeding of the cells causes unpredictable, inhomogeneous gaps between the individual cells as the U87 cell line used in this study does not grow in homogeneous monolayers but tends to cluster in bulks. Thus, in one sample there can be areas with a very high cell density and areas with a very low cell density. To exclude a bias coming from selection of distinct locations, the imaged positions were randomly chosen. Consequently, very high local differences in the cell density can occur. However, the temporal development of connected cells (see Figures 2, 3) reveals that the cell-to-cell connectivity increases, although the cell density does not change significantly. Therefore, we assume that the cell density has only a subordinate role in the TNT establishment. Nevertheless, it would be beneficial to have similar cell-to-cell distances when studying the role of TNTs in stress conditions.

## Conclusion

In this work, we introduced a method to examine TNT networks *in vitro* in a quantitative and qualitative manner with the aim to obtain a better understanding of cellular communication networks. Furthermore, we figured out that the cellular communication via TNTs is influenced by radiation in U87 glioblastoma cells. This could mean that there may be an additional mechanism which causes the irradiated cells to form TNTs faster and more frequently than normal. It might be that irradiated cells release signal molecules into the medium which can lead to an increased TNT formation by filopodia growth. Additionally, our results show that irradiated U87 cell populations have more *complex* connections consisting of several TNTs as compared to non-irradiated cell populations after 24 h. This could signify that the irradiated cells strengthen their TNT network more intensively than non-treated cells. This probably indicates an increased communication via TNTs with the idea that the more TNTs, the more cargoes can be transferred at the same time. However, this hypothesis remains to be proven. After 72 h of incubation, our results suggest that the most features of the TNT network are the same again for both irradiated and non-irradiated samples.

For obtaining an even better understanding of how cellular communication via TNTs is involved or activated after exposure to radiation, it is necessary to perform live-cell imaging videos of TNT formations in irradiated and non-irradiated cells. Here, cell-tracking of single cells would be beneficial. It is also important to find out whether cell dislodgement or division leads to the formation of one single TNT or to several densely packed TNTs. Furthermore, it is essential to identify the transferred cargoes along the TNTs, since this would provide a better understanding of the interfering mechanisms of cell-to-cell communication and would be the evidence that there is indeed an exchange of information via TNTs after irradiation.

Overall, intercellular communication via TNTs seems to play an important role in the response of glioblastoma cells to radiation. A better understanding of the mechanisms and the biological functions behind these communication networks could help to improve the treatment of these aggressive tumors in the future.

## Data Availability Statement

All datasets presented in this study are included in the article/Supplementary Material.

## Author Contributions

NM designed the study, performed the experiment and analysis, discussed the data, and wrote the manuscript. JR gave the idea and designed the study, discussed the data, and wrote the manuscript. Both authors contributed to the article and approved the submitted version.

## Conflict of Interest

The authors declare that the research was conducted in the absence of any commercial or financial relationships that could be construed as a potential conflict of interest.

## Abbreviations

DSB: DNA double strand break
TNT: tunneling nanotube.
