# Supplementary material for: Influence of α-Particle Radiation on Intercellular Communication Networks of Tunneling Nanotubes in U87 Glioblastoma Cells
Source: Front Oncol. 2020 Sep 4;10:1691. doi: 10.3389/fonc.2020.01691 (PMC7509401; doi:10.3389/fonc.2020.01691)
Supplement: Supplementary file 1 [file Data_Sheet_1.pdf]

# *Supplementary Material*

## 1 Supplementary Tables

| | Time after Irradiation [h] | 2 TNTs per Connection | 3 TNTs per Connection | 4 TNTs per Connection |
|---|---|---|---|---|
| Sham | 1 | 0.27 ± 0.08 | 0.091 ± 0.067 | 0.054 ± 0.032 |
| | 6 | 0.17 ± 0.04 | 0.085 ± 0.029 | 0.094 ± 0.015 |
| | 24 | 0.19 ± 0.013 | 0.041 ± 0.021 | 0.049 ± 0.004 |
| | 72 | 0.21 ± 0.011 | 0.11 ± 0.03 | 0.047 ± 0.008 |
| Irradiated | 1 | 0.19 ± 0.05 | 0.082 ± 0.021 | 0.032 ± 0.024 |
| | 6 | 0.16 ± 0.022 | 0.10 ± 0.03 | 0.057 ± 0.014 |
| | 24 | 0.20 ± 0.012 | 0.075 ± 0.009 | 0.060 ± 0.006 |
| | 72 | 0.16 ± 0.008 | 0.11 ± 0.016 | 0.060 ± 0.0015 |

**Supplementary Table 1:** Number of cell-to-cell connections consisting of 2, 3 or 4 TNTs normalized to the total number of found connections. Mean values ± SEM are shown.

## 2 Supplementary Figures

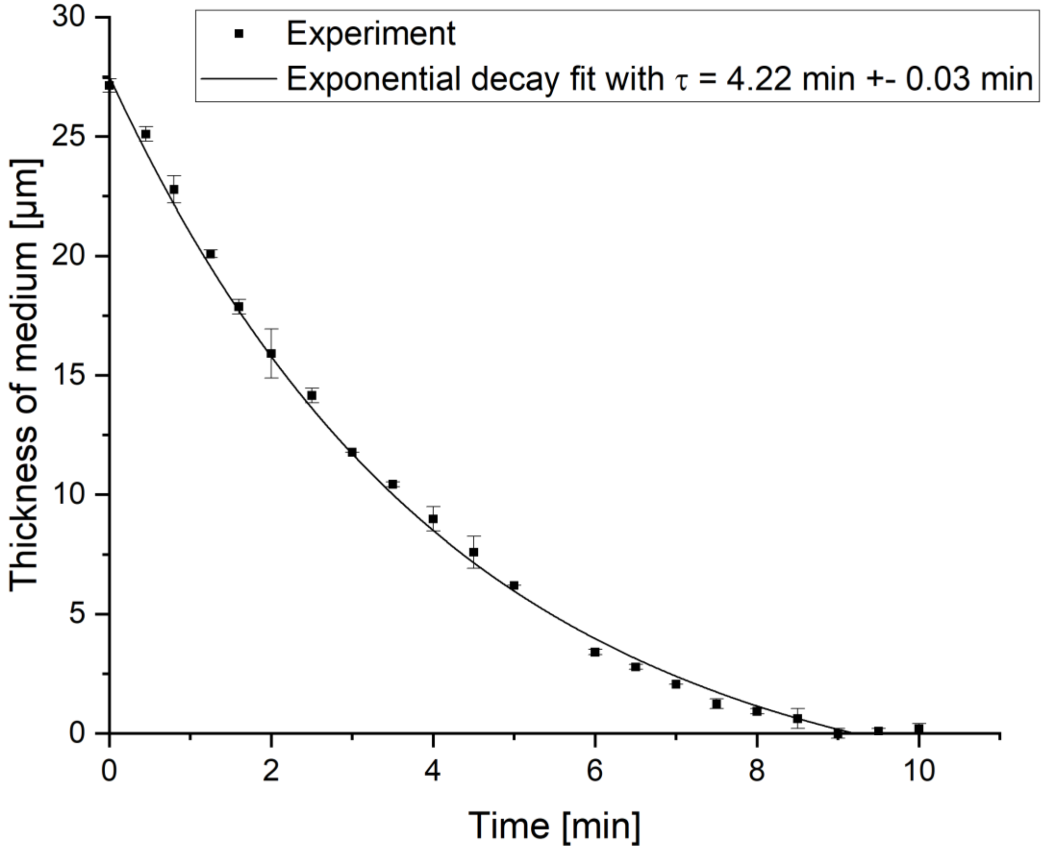


**Supplementary Figure 1:** Measurement of the medium evaporation on a cell seeded cover glass during irradiation over time. The thickness of the medium layer was determined by weighting of the cell seeded cover glass and assuming of a uniformly distributed medium layer. The experiment was conducted at a room temperature of 19 °C and a humidity of 40 %. The cell seeded cover glass was cultured in the incubator at a temperature of 37 °C (100 % humidity, 5 % CO2) before the experiment.

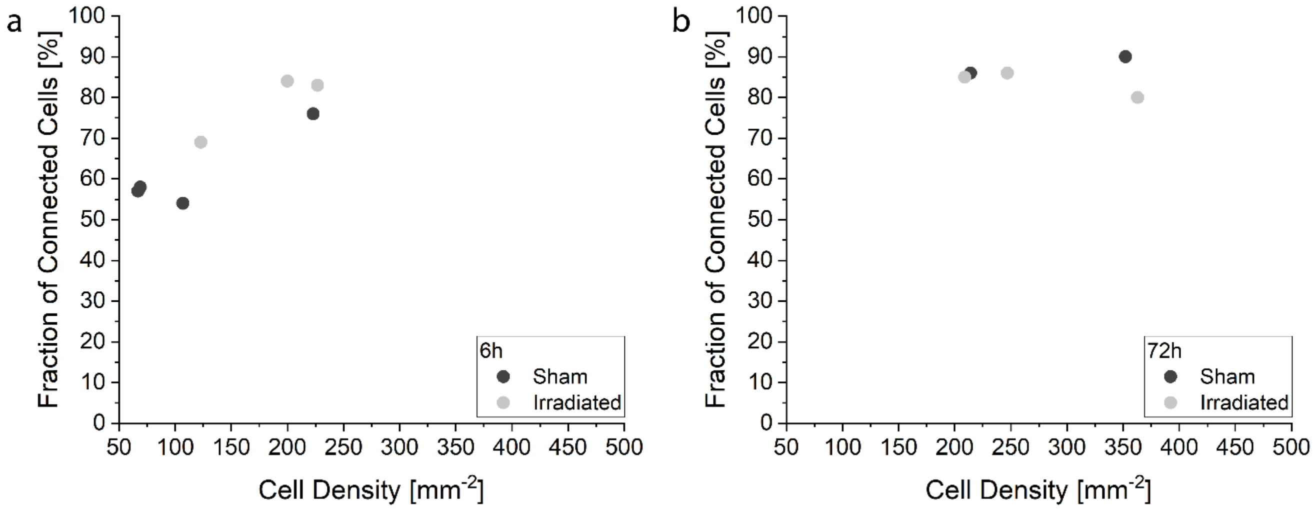


**Supplementary Figure 2:** Scatter plots for incubation times 6 h (a) and 72 h (b) showing the fraction of connected cells in dependence of the cell density for each sample.

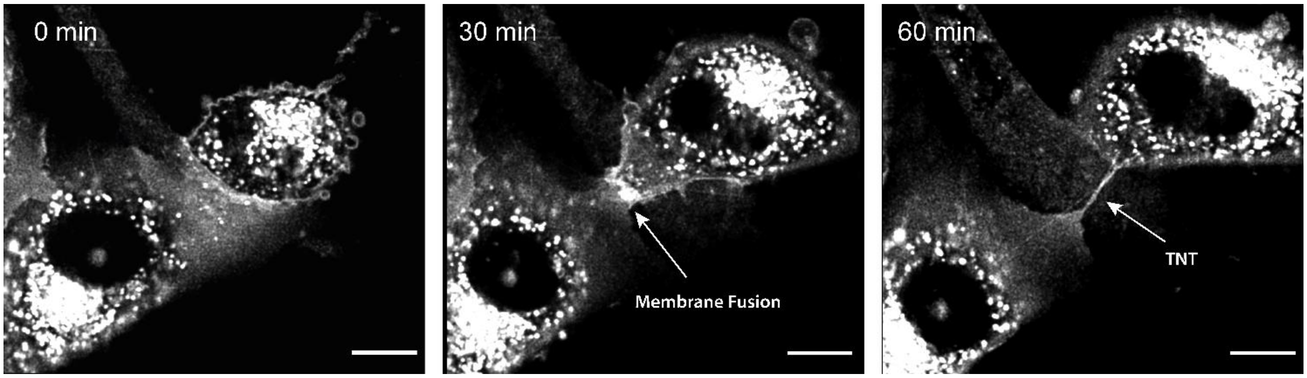


**Supplementary Figure 3:** Confocal video microscopy images of a TNT formation in U87 cells labeled with CellMask™ orange plasma membrane stain. The TNT was formed within one hour via cell dislodgment. Scale bars: 10 µm.

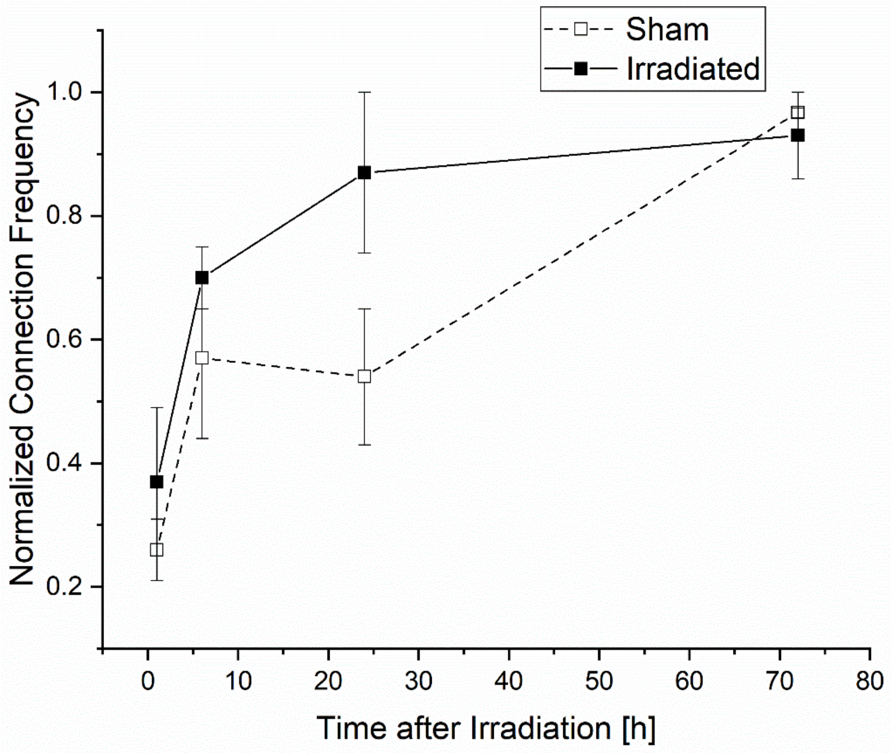


**Supplementary Figure 4:** Temporal development of the connection frequency determined by summing up all found connections and normalized to the total number of cells. Shown are mean values ± SEM.
